# Solid medical waste: a cross sectional study of household disposal practices and reported harm in Southern Ghana

**DOI:** 10.1186/s12889-017-4366-9

**Published:** 2017-05-18

**Authors:** Emilia Asuquo Udofia, Gabriel Gulis, Julius Fobil

**Affiliations:** 1 Department of Biological, Environmental and Occupational Health Sciences, School of Public Health, University of Ghana, Legon, Ghana; 2 Department of Community Health, School of Public Health, University of Ghana, Legon, Ghana; 3 Unit for Health Promotion Research, University of Southern Denmark, Esbjerg, Denmark

**Keywords:** Healthcare waste, Medical waste, Disposal, Community, Ghana, Waste management

## Abstract

**Background:**

Solid medical waste (SMW) in households is perceived to pose minimal risks to the public compared to SMW generated from healthcare facilities. While waste from healthcare facilities is subject to recommended safety measures to minimize risks to human health and the environment, similar waste in households is often untreated and co-mingled with household waste which ends up in landfills and open dumps in many African countries. In Ghana, the management of this potentially hazardous waste stream at household and community level has not been widely reported. The objective of this study was to investigate household disposal practices and harm resulting from SMW generated in households and the community.

**Methods:**

A cross-sectional questionnaire survey of 600 households was undertaken in Ga South Municipal Assembly in Accra, Ghana from mid-April to June, 2014. Factors investigated included socio-demographic characteristics, medication related practices, the belief that one is at risk of diseases associated with SMW, SMW disposal practices and reported harm associated with SMW at home and in the community.

**Results:**

Eighty percent and 89% of respondents discarded unwanted medicines and sharps in household refuse bins respectively. A corresponding 23% and 35% of respondents discarded these items without a container. Harm from SMW in the household and in the community was reported by 5% and 3% of respondents respectively. Persons who believed they were at risk of diseases associated with SMW were nearly three times more likely to report harm in the household (OR 2.75, 95%CI 1.15–6.54).

**Conclusion:**

The belief that one can be harmed by diseases associated with SMW influenced reporting rates in the study area. Disposal practices suggest the presence of unwanted medicines and sharps in the household waste stream conferring on it hazardous properties. Given the low rates of harm reported, elimination of preventable harm might justify community intervention.

## Background

Medical waste refers to any discarded solid material generated from activities involving health protection, medical diagnosis, treatment, scientific research, as well as dental and veterinary services [1–3]. Medical waste is a concern due to its ability to cause injury, potential for disease transmission and environmental pollution [4]. CDC and EPA recognize that medical waste that harbors pathogens, blood and blood products, contaminated human or animal tissue or body parts and sharps, as infectious [5]. Although hospitals are the major producers of medical waste, the increasing tendency for home healthcare, early discharge from surgical care and home management of illnesses indicates that the concerns of medical waste cannot be limited to the hospital environment [6–9]. In Ghana, healthcare waste is generated within households where healthcare practitioners and/or family members provide care to a patient. However, waste material from healthcare activities in the household are not handled nor treated in a similar manner as that produced in hospitals [10]. The technology applied in hospital settings cannot be used safely in households. Disposal practices regarding waste generated from healthcare activities in households have not been widely reported in Ghana. Additionally, there is no published report of harm associated with this potentially hazardous waste stream in households and the community. There is also no policy that specifically addresses household medical waste disposal.

In the United States, it is estimated that 1 in 12 households use a syringe for the treatment of diabetes, migraines, allergies, infertility, arthritis, osteoporosis, HIV, hepatitis among other conditions. This inadvertently increases the use of injectable medications and the devices often end up in trash [6]. Patients can seek early discharge from hospital admissions to reduce costs or wish to return to their families and opt to be cared for at home. Where medical care is provided in the household, medical devices used are ultimately discarded in household bins [4, 10]. Medicines left over from previous use or expired ones are often discarded in household bins [11, 12]. In a study in Ghana, it was reported that over 75% of respondents discarded pharmaceutical waste (left over and expired medicines) in household refuse that eventually ended up in landfills or dump sites [13]. A study on clinical waste management in an HIV/AIDS home based care program in Botswana, reports that stigma associated with red medical waste bags discouraged some primary caregivers (individuals or family members who stay with a client) from conveying the waste to the clinic. In that study, waste was reportedly discarded in council bins, in a nearby bush, burnt or buried clandestinely [14].

A major safety concern is that of transmission of blood borne viruses such as HIV, Hepatitis B and Hepatitis C viruses, through needle stick injuries. A recent analysis of 21 studies on community acquired needle stick injuries (CANSIs) and their outcome(s) documents six cases of blood borne virus transmission attributed to CANSIs [15]. All patients developed hepatitis, but no cases of HIV transmission were documented [15]. In most of the infected cases, delayed or absent immunoprophylaxis appears to have enhanced vulnerability. There are three reported cases of Hepatitis B infection from Spain [16]; Tbilisi, Georgia [17]; and Australia [18].

Burning waste generated from healthcare activities in the household contributes to environmental pollution by the release of toxic air emissions. Burning plastics widely used for disposable materials and chlorinated materials are major sources of dioxins. Dioxins are a common term for chemical compounds consisting of chlorinated dibenzo-furans and dibenzo-dioxins known to be toxic and to have carcinogenic potential [19]. However, the relatively small quantities of waste generated from healthcare activities in households are likely to emit lower concentrations compared to other sources of these materials. Leaching of toxic heavy metals and chemicals from solid medical waste (SMW) into the soil occurs in poorly engineered landfills and dump sites [20]. These are ultimately absorbed in the food chain and consumed by man. In addition, leachate can percolate through the soil and contaminate surface and groundwater supplies posing threats to human health by consumption of unsafe water. Although, the contribution of leachate from home generated solid medical waste can be considered minimal relative to hospital waste, it poses a potential hazard to the environment and human health. The consequences of inadequate management of solid medical waste are not limited to patients, their relatives, and health care workers but affects waste workers, scavengers, and the unsuspecting public [2, 21, 22].

The present study aimed to (i) investigate disposal practices relating to waste generated from healthcare activities in the household and (ii) document reported harm associated with this waste in Ga South Municipal Assembly of the Greater Accra Region.

## Methods

### Study area

The study area is Ga South Municipal Assembly, an administrative unit (district) in the Greater Accra Region (GAR), Ghana. It has a population of 411,377 representing 10.3% of GAR’s population of 4,010,054 according to the 2010 national population census [23]. There are 100,701 households with an average household size of 4 (Unpublished document, GSS, 2014). It is predominantly urban (88.7%); the dwellers are mainly artisan, distributive traders, handcrafters, civil servants and industrial workers.

### Research design

A household questionnaire survey was conducted using a cross sectional design which permits an assessment of factors and outcomes of interest simultaneously in a representative sample of the target population.

### Sampling

A sample of 384 households was obtained using the expression, n = Z^2^α*pq*/L^2^ where the proportion of households reporting harm due to SMW, *p* was assumed to be 50% [24], allowable error, *L* was 5%, *q* being *1-p*, and Zα being the standard normal deviate with a value of 1.96 [25]. We allowed for a non-response rate of 54% based on previous literature and rounded up to 600 households [26]. The households were selected by multi-stage sampling of sub-districts and localities in the study area. An eligible respondent was aged 18 years or older to ensure that they were adults mature enough to participate in the study and did not require assent from parents to respond. They were also expected to be well informed about the household or had lived for at least one month with the household. This was considered long enough to enable the respondent be familiar with housekeeping activities in the household including waste management. The questionnaire was administered to one eligible respondent in a household and where there was more than one was present, the respondent was selected by ballot.

### Research instruments

The tool was a structured questionnaire, purpose designed by the researcher and pre-tested in five households before use. It was pre-tested to ensure that the questions were uniformly understood and there were no ambiguities. Modifications were made to the questionnaire following the pre-test. It was also translated into a local dialect and back translated to ensure that the meaning was not affected in the possible event that respondents preferred to conduct the interview in the local dialect. The questionnaire was structured to capture household characteristics grouped under four domains. Socio-demographic characteristics included age, gender, marital status, religion, ethnicity, level of schooling and occupation. Medication related information included visits to a chemical vendor shop, a health facility (clinic, health centre, hospital), conditions for the visit and prescriber for medication bought from the chemical shop vendor. SMW disposal practices referred to the way different types of SMW were discarded. The types of medical waste were: unwanted or expired medication referring to left over medicines which were no longer used in the household or which had attained the expiry date respectively; sharps which are items that can cut or pierce the skin to cause injury; soiled items referred to materials contaminated with pus and serum; blood soaked items were materials stained with blood and separate questions were asked about disposal of placenta. Households reporting harm associated with SMW were obtained by direct questions regarding harm in the household and in the community (outside the household). In addition, respondents were asked to list diseases associated with disposal of SMW and specific items considered a problem in the community. For each list, respondents were allowed to list five responses, though majority listed three responses or less. Therefore, the number of responses differed from the number of respondents. In the respective tables, both the proportion of responses and proportion of respondents were presented.

### Data collection

Data collection was conducted from mid-April to June, 2014. Permission was sought from the household heads for questionnaire administration in the household and a written, individual informed consent obtained from the eligible respondent. The language of communication was English or the local dialect where this was preferred. Questionnaire administration was conducted mainly in the evenings to maximize the chances of meeting families at home and lasted approximately 30 min for each respondent.

### Data processing and analysis

Descriptive and inferential analysis were done using Statistical Package for Social Sciences (SPSS) version 22 for Windows software (SPSS, Inc., Chicago, IL). Respondents’ ages were grouped and presented as simple frequencies and proportions along with other sociodemographic variables. The dependent variable (harm associated with SMW) and independent variables (socio-demographic characteristics, belief that one is at risk of having diseases associated with SMW and having been affected by diseases associated with SMW) were dichotomized with ‘1′ representing the reference category and ‘2′ representing the risk category. Associations between the dependent and independent variables were evaluated using the Pearson’s chi-squared test and binary logistic regression analysis. Results were reported as Odd’s ratios (OR) with the 95% confidence interval (95% CI). The level of statistical significance was set at *p* ≤ 0.05.

## Results

### Socio-demographic characteristics

A total of 600 households were included in the survey. Respondents were predominantly female 411 (68.5%), of the Ga ethnic group 195 (32.5%), married 341 (56.8%), Christians 530 (88.3%) and engaged in trading 155 (25.8%). More than half of the respondents had secondary level education 323 (53.8%) (Table 1).

**Table 1:** Sociodemographic characteristics of survey respondents, Ga South Municipal Assembly, 2014

Variable (Number)	Number (%)
Gender	
Male	189 (31.5)
Female	411 (68.5)
Age in years	
18–24	69 (11.5)
25–29	111 (18.5)
30–34	88 (14.7)
35–39	104 (17.3)
40–44	71 (11.8)
45+	157 (26.2)
Ethnicity	
Akan	126 (21.0)
Ga/Dangme	195 (32.5)
Fante	72 (12.0)
Twi	74 (12.3)
Ewe	90 (15.0)
Others^a^	41 (6.8)
Marital status	
Never married	141 (23.5)
Married	341 (56.8)
Separated/Divorced	41 (6.8)
Cohabiting	14 (2.3)
Widow(er)	63 (10.5)
Religious Affiliation	
Christianity	530 (88.3)
Islam	57 (9.5)
Others^b^	13 (2.1)
Occupation	
Agriculture	80 (13.3)
Artisanship/Transport/Services	93 (15.5)
Civil servants	104 (17.3)
Trading	155 (25.8)
Manual labour/Others^c^	33 (5.5)
Pensioner	20 (3.3)
Unemployed	115 (19.2)
Highest level of schooling	
Primary	77 (12.8)
Secondary	323 (53.8)
Vocational/Technical/Other	29 (4.8)
Post-basic/Tertiary	107 (17.8)
Not applicable	64 (10.7)

^a^ Mole Dagbon 1 (0.2), Northern tribes 23 (3.8), Hausa 17 (2.8), Non-Ghanaian 2 (0.3); ^b^ Traditional African religion 11 (1.8), not specified 2 (0.3); ^c^ Lotto player 1(0.2), coach 1(0.2), freelancer 1(0.2), footballer 1(0.2), clergy men 2 (0.3)

### Medication related information and disposal practices

Respondents’ contact with health and supportive systems and their medication related practices were assessed for the two weeks preceding the survey (Table 2). Chemical shop visits were made by 250 (41.7%) respondents mainly for headaches or stress 131 (21.8%), followed by abdominal discomfort/upset 40 (6.7%) and febrile illness 28 (4.7%). Most respondents engaged in self-medication 133 (22.2%). There were fewer visits 99 (16.5%) to healthcare facilities (clinics, health centres, hospital) within the same period. Acute illnesses (mainly fevers) and pregnancy accounted for the majority of health facility visits involving 22 (3.7%) and 19 (3.2%) respondents respectively.

**Table 2:** Medication practices of household in the preceding two weeks, Ga South Municipal Assembly, 2014

	Number (%)
Member visited chemical shop vendor	
Yes	250 (41.7)
No/Not sure	350 (58.3)
Reason for visit to chemical shop vendor	
Stress/headaches	131 (21.8)
Abdominal discomfort/upset	40 (6.7)
Febrile illness	28 (4.7)
Pain	18 (3.0)
Injury/burn	8 (1.3)
Cough/cold	8 (1.3)
Drug refill	7 (1.2)
Others^a^	11 (1.8)
Not applicable	349 (58.2)
Person who prescribed medication	
Self	133 (22.2)
Recently prescribed by doctor	66 (11.0)
Prescribed earlier by doctor	12 (2.0)
Chemical vendor/Other^b^	39 (6.5)
Not applicable	350 (58.3)
Member visited health facility	
Yes	99 (16.5)
No/Not sure	501 (83.5)
Reason for visit to health facility	
Not aware	29 (4.8)
Pregnancy	19 (3.2)
Scheduled immunization	10 (1.7)
Acute illness	22 (3.7)
Chronic illness	8 (1.3)
Accidents	6 (1.0)
Others^c^	4 (0.7)
Not applicable	501(83.5)

^a^ Rashes/boil 3(0.5), anti-helminthic 2(0.3), genital itch/discharge 2(0.3), loss of appetite 1(0.2); ^b^ Friend/Neighbour 1(0.2); ^c^ Check up 2(0.3), eye infection 1(0.2), rashes 1(0.2)

Unused medications and sharps waste were mostly discarded in household bins (Table 3). Unwanted medicines were often discarded in a container 341 (56.8%) or loosely 139 (23.2%) in the household bin (Table 4). Thirty four respondents (5.6%) reported giving unused medicines out to other people who may needed them. Disposal of sharps followed a similar pattern with the majority discarding sharps in a container 321 (53.5%) or loosely 210 (35%) in the household bin. Soiled items (*n* = 582) were often wrapped and discarded in the dustbin 409 (68.2%) or burnt/buried 114 (19.6%). Blood soaked items (*n* = 592) including sanitary pads were discarded in the dustbin 240 (40.6%) and also burnt/buried 200 (33.8%) (Table 3).

**Table 3:** Disposal practices by waste category in Ga South Municipal Assembly, 2014

Waste category	Dustbin *N* (%)	Burn/Burial/Others *N* (%)	Not applicable *N* (%)
Sharps waste (*n* = 600)	534 (89.0)	56 (9.3)	10 (1.7)
Unwanted medicines (*n* = 600)	481 (80.2)	113 (18.8)	6 (1.0)
Soiled items (*n* = 582)	454 (78.0)	116 (19.9)	12 (2.1)
Blood soaked items (*n* = 592)	250 (42.2)	240 (40.6)	102 (17.2)

*N* (*n*) = number of respondents, (%) = percentage; Others in each category: sharps waste = drop anywhere - 2, bush −5; unwanted medicines = drop in the gutter - 1, toilet - 1, keep them - 2, bush - 7, give out to others - 34; soiled items = bush - 1, toilet - 1; blood soaked items = toilet – 35, bush – 5

**Table 4:** Disposal of some categories of solid medical waste in refuse bins (*n* = 600 households)

Item	Household Bin		Roadside Bin
	Wrapped/In container	Loose	
Soiled items	409 (68.2)	45 (7.5)	-
Unwanted/expired medicines	341 (56.8)	139 (23.2)	1 (0.2)
Sharps waste	321 (53.5)	210 (35.0)	3 (0.5)

Status for blood soaked items was not specified

Table 5 shows other disposal practices in relation to SMW. When asked if old medicine or disinfected containers were re-used or sold, less than 4 % responded affirmatively: 23 (3.8%) and 6 (1.0%) respectively. Forty seven respondents (7.8%) brought placentae home following delivery which was often buried in the ground.

**Table 5:** Other disposal practices related to solid medical waste, Ga South Municipal Assembly, 2014

Practices	Number (%)
Birth related	
Placenta brought home after delivery	47 (7.8)^a^
Placenta buried	46 (7.7)^a^
Informal reuse of containers	
Ever sold old medicine bottles	6 (1.0)^a^
Re-use old medicine or disinfectant containers	23 (3.8)^a^
Nature of household bin	
Bucket with(out) lid	197 (32.9)
Sack/cellophane bag	142 (23.7)
Basket/basin/carton	73 (12.2)
Standard bin	116 (19.3)
Gallon	21 (3.5)
Pit	38 (6.3)
None (nearby bush)	9 (1.5)
No response	4 (0.7)
Waste is removed from home by	
Burial in a pit in the compound	14 (2.3)
Burning in the compound	94 (15.7)
Carried to a communal bin	258 (43.0)
Picked up by a refuse truck	208 (34.7)
Dump in a nearby bush	21 (3.5)
Other^b^	2 (0.3)
No response	3 (0.5)
Waste is conveyed to disposal point by	
Children aged <10 years	4 (0.7)
Adolescents aged 10–19 years	161 (26.8)
Adults aged ≥20 years	402 (67.0)
Not applicable	19 (3.2)
No response	14 (2.3)

^a^ Yes responses only (placenta buried: one respondent did not indicate where it is disposed); ^b^ Burning outside the compound 1(0.2), dump in the gutter 1 (0.2)

Different items were used to store waste in the households with the majority using a bucket with/without a lid 197 (32.9%), a sack or cellophane bag 142 (23.7%) followed by standard bins provided by waste collection companies 116 (19.3%). From the households, waste was mostly carried to a communal bin 258 (43.0%) and picked up by a refuse truck 208 (34.7%). The conveyance to the point of final disposal was done by adults in two-thirds of households followed by adolescents 161 (26.8%).

### Reported harm associated with SMW

Twenty nine respondents (4.8%) reported harm associated with SMW in the household, while 16 respondents (2.7%) reported harm associated with SMW in the community where they reside. Reported harm from SMW was mostly due to used razor blades 11 (1.8%). Two households reported that more than one individual was affected, compared to one individual in the remaining households. In the community, reported harm was mostly attributed to broken medication bottles/glass 9 (1.5%) and only one individual was reportedly harmed in each affected household (Table 6). Table 7 shows that in 8 (1.3%) households, the injury occurred in the open field. These 8 households represented half of all households (*n* = 16) that reported harm from SMW in the community.

**Table 6:** Items causing harm from disposal of solid medical waste, Ga South Municipal Assembly, 2014

Item causing harm	At home Number (%)	Individuals affected	In the community Number (%)	Individuals affected
Needle (with or without syringe)	7 (1.2)	8	4 (0.7)	4
Broken medication bottle/glass	10 (1.7)	10	9 (1.5)	9
Used razor blade	11 (1.8)	13	2 (0.3)	2
Other specify	1 (0.2)^a^	1	1 (0.2)^b^	1
Not applicable	571 (95.2)		584 (97.3)	

^a^ Medication (tablets), ^b^ Sharp/cutting instrument

**Table 7:** Places where injury from solid medical waste occurred in the community, Ga South Municipal Assembly, 2014

Place injury occurred	Number (%)
Along the road	4 (0.7)
Near a refuse dump	3 (0.5)
In an open field	8 (1.3)
Other specify	1 (0.2)
Not applicable	584 (97.3)

### Diseases associated with SMW

Each respondent was asked about diseases which can be associated with exposure to body fluids or items in SMW. The first three responses were summarized in Table 8. The top three diseases listed were tetanus (48%), HIV/AIDS (45%) and tuberculosis/chronic cough (23%). Table 9 summarizes SMW considered a problem in the community from the multiple responses provided. Excluding offensive waste, used/contaminated blades ranked first (13%) followed by broken medication bottles (10%).

**Table 8:** Diseases associated with disposal of solid medical waste in households as suggested by respondents, Ga South Municipal Assembly, 2014

Diseases	Number of responses	% of responses *N* = 806	% of respondents *N* = 600
HIV/AIDS	267	33%	45%
Tetanus	287	36%	48%
Tuberculosis/Chronic cough	139	17%	23%
Hepatitis B	27	3%	5%
Mild cough/Catarrh	14	2%	2%
Skin rashes/infections	7	1%	1%
Diarrhoeal disease	12	1%	2%
Other infectious/febrile diseases	39	5%	7%
Other not categorized	14	2%	2%

**Table 9:** Discarded solid medical waste items considered a problem in the community, Ga South Municipal Assembly, 2014

Items	Number of responses	% of responses *N* = 387	% of respondents *N* = 600
Broken medication bottles	59	15%	10%
Used condoms^b^	112	29%	19%
Soiled diapers^b^	67	17%	11%
Used sanitary pads^b^	51	13%	9%
Used blades	75	19%	13%
Needles/syringes	10	3%	2%
Others^a^	13	3%	2%

^a^ Unclear how to categorize. ^b^ Not generally considered SMW but included as they may contain body fluids or excrement which unless otherwise determined were assumed potentially infectious under the typical conditions of disposal

### Factors associated with harm from SMW in the household

Based on the chi-square test, four factors were associated with households reporting harm from SMW. These were gender and educational status of the respondent, believing that s/he is at risk of having a disease associated with SMW, and being affected by a disease associated with SMW. After multivariable analysis (Table 10), only one factor was independently associated with harm related to SMW namely, believing oneself to be at risk of having a disease associated with SMW. Respondents who believed themselves to be at risk of a disease associated with SMW were nearly three times more likely to report harm compared to those who did not consider themselves at risk (OR 2.75, 95% CI 1.15–6.54).

**Table 10:** Factors associated with harm from home disposal of solid medical waste, Ga South Municipal Assembly, 2014

Variables (risk group)	OR	*p*-value	95% CI	
			Lower limit	Upper limit
Had a disease associated with SMW (Yes)	3.056	0.091	0.835	11.178
Believes s/he is at risk of having a disease associated with SMW (Yes)	*2.746*	*0.023*	*1.152*	*6.543*
Education (None/Basic)	2.158	0.087	0.893	5.213
Gender (Male)	0.435	0.141	0.143	1.319

*OR* Odds ratio, *95% CI* 95% confidence interval, statistical significance was set at *p*<0.05 (italic)

## Discussion

### Waste management practices

The results indicate that most respondents practiced self-medication, had some contact with a facility in the preceding two weeks and have various options for discarding SMW. Comparable with previous studies, the bulk of SMW was reportedly deposited in the household bin in containers or loosely, including sharps [4, 27]. Sharps were a major concern in the community and accounted for harm associated with SMW both in and outside the household. Mixing SMW with general waste in the household suggests cross contamination of household waste, especially when SMW was discarded loosely and if pathogens were present. When SMW is mixed with general waste in healthcare facilities, the whole load is assumed and treated as contaminated or potentially infectious [28, 29]. It is therefore expected to be treated to minimize risk to the environment prior to final disposal. However, SMW generated in households is not treated before final disposal at the landfill or designated dumpsites for municipal solid waste.

The largest proportions of SMW disposed in the household bin were unwanted medicines and sharps. The practice of disposal of unwanted medicines in household bins has been reported in the UK, Kuwait and Lithuania [30]. About 25% of remnant household pharmaceutical waste was disposed with municipal solid waste in Germany and Austria [31]. In one study in Nigeria, all respondents reported discarding unused medicines in the household bin [32]. The ultimate disposal of household refuse containing pharmaceutical agents in improperly engineered landfills is a cause for concern. It has been reported that low dose environmental exposure to some pharmaceuticals contributes to ecotoxicological effects in aquatic and terrestrial life [33]. Leachate from landfills and other dumpsites containing SMW can contaminate surface and ground water resources which serve as sources of drinking water. Agricultural food crops can absorb active pharmaceutical ingredients leading to unintentional, often long term and intermittent exposure in humans [11].

The survey showed that 23% of medicines are discarded loosely in household bins. Within a household, loosely discarded medicines may be acquired by unsupervised young children and might result in acute poisoning [11]. Although only one case of acute poisoning was reported in this study, the high proportion of households discarding unwanted medicines in the household bin raises safety concerns. In contrast, Sweden has had a take-back program for unwanted medicines in place for decades and an estimated 73% of Swedish return unused medicines to pharmacies. These are eventually incinerated at high temperatures and the residue deposited in specified landfills [30]. Consequently, environmental contamination with pharmaceutical products is less likely to cause much concern in Sweden.

A larger proportion of households (35%) discarded sharps loosely (not placed in a container) in household bins. This could pose a danger to unsupervised young children or other unsuspecting household members who come in contact with the waste containing contaminated sharps. Young children may get pricked from used needles discarded around the household or within the community although our survey showed that the proportion of households affected was small (<5%). The low rate of reported harm associated with SMW is consistent with earlier studies in Kenya [34] and USA [35]. Furthermore, the use of non-rigid containers (cellophane bags) for temporary storage of household solid waste containing SMW by some households is a potential source of exposure. Persons charged with removing the waste from the household are at risk of sharps-related injury. In spite of the low rates reported in this study, the implied cost of required medical care is potentially high and can place a strain on household finances. In United States, the median cost of an emergency department evaluation and treatment for a community acquired needlestick was estimated at $575 and higher if a referral was made to an infectious disease physician [35]. Expectedly, the costs of care may be lower in most developing countries, or partly absorbed by national health insurance, but most households will not be able to afford it. Furthermore, if there is no perceived risk by household members, the event may go unreported. This study showed that those who reported an event of harm were more likely to be those who perceived themselves at risk of diseases associated with SMW. Aside from the possibility of transmission of pathogens resulting from inoculation injuries, other difficulties with medical costs and care, the lack of concentration, anxiety and emotional distress preceding and after laboratory investigations cannot be overlooked [36]. The anxiety about potential transmission of blood borne viruses can be stressful over the period required to exclude infection [37].

Broken medication bottles were some of the SMW items that were considered a problem within the community and contributed to 2% of reports at home and in the community. Broken glassware causes cuts in the skin which allow penetration of dirt and pathogens through the non-intact skin predisposing to infections such as tetanus. This was one of the diseases respondents listed in association with SMW, indicating some level of awareness. Although the proportion of persons affected was small, elimination of preventable hazards from SMW should be aimed at to ensure a safe environment.

Burning household waste at low temperatures as seen with back yard burning in shallow pits produces incomplete combustion of materials, toxic air emissions and smoke. Persistent free radicals, heavy metals and poly aromatic hydrocarbons (PAHs) are released by burning plastics. These are found in residue solid ash and particulate soot which is harmful to respiratory health when inhaled [38]. Our survey showed that 16% of households burnt household waste including SMW. Burning plastic chlorinated materials which are common in SMW also has the potential to expose household members to dioxins which are carcinogenic, although exposures are probably minimal compared to exposures attributed to poorly functioning medical waste incinerators.

### Benefits of the study

Knowledge about disposal methods could be helpful in accounting for possible losses in the estimates of SMW generated from residential dustbins. Our findings suggest that waste stream analysis on samples taken from the household bin in similar settings would recover most unwanted medicines and sharps, but a considerable proportion of blood soaked items would be lost since these are also burnt and buried. Pathological waste such as placenta from home births is unlikely to be recovered from sampling the household bin because it was buried. The low reporting rate of households associated with harm from SMW at home or in the community, in spite of potentially hazardous disposal practices is unclear but consistent with earlier studies. Potential explanations include low risk perception as well as the fact that the presence of SMW does not necessarily translate to harm. The reporting rates for harm associated with waste generated from healthcare activities in households could serve as a proxy indicator of the risk associated with this type of waste in the community.

### Future directions and policy

The pharmaceuticals in household SMW should be diverted for environmental protection and prevention of drug resistance. Safe handling and disposal of sharps waste to reduce potential harm is crucial. Specific policy guidance is required and should include the responsibilities of household members, healthcare workers, district assemblies, waste management companies and other stakeholders. Sanctions for non-compliance should be specified and known to the public to enhance compliance. Public implementation guidelines should include how to safely segregate waste categories at source. For source segregation to be successful in the present context, it would rely heavily on intensive public education, as well as adoption and monitoring of public collection points because door-to-door collection may not be feasible in all areas. Monitoring and enforcement at household level has been considered impractical, which implies that individuals must be self-motivated to segregate SMW or be otherwise incentivized. The availability of community health workers who can make home visits, reinforce appropriate information and lend technical support when trained, presents a potential opportunity, if given the necessary logistic support and motivation. Other specific recommendations are discussed in the sections following and summarized in schema at the end.

### Recommendations at community level

Public education about the handling and disposal of waste generated from healthcare activities in the household should be provided. Community members have a right to know about hazardous properties of waste generated from healthcare activities in the household and how to safely dispose of it. Communication should be done in local dialects on electronic media as well as in English. Regarding harm from SMW, community members need to know what should be done in the event of harm, how and where to report such events. The reporting pathway for the public should be short to improve reporting rates. Community clinics and health centers are frontline healthcare facilities which can be supervised by infection control units at district hospitals. Within communities, traditional leaders, local chiefs and queen mothers play a vital role in community mobilization and information dissemination. They can mobilize women groups, landlords associations, and youth groups to promote safe practices.

### Recommendations at district level (Municipal Assembly)

In pursuit of segregation at source as a potential management option, waste quantification is required at regional level to plan for storage capacity and equipment at source (households or other sources) and at potential collection points. This can be undertaken by waste management departments of district assemblies, in collaboration with research based universities, other public and private stakeholders in waste management. Landfill studies are required in areas where mixed waste streams existed and in cells specific for healthcare waste to detect hazardous effects in the environment, particularly where attenuation processes may have been overwhelmed. In such areas, stringent measures are needed to limit further pollution to the environment and ultimately protect public health.

To reduce backyard burning, particularly of offensive waste, frequent and timely collection is required. Sanitary waste in particular tends to leave offensive odors, encouraging households to resort to burning them as a convenient means of disposal. The frequency of collection is dependent on availability of treatment, transfer stations and/or final disposal sites so that waste management companies can make their rounds in shorter cycles. It also requires that service consumers pay agreed collection rates.

### Recommendations to the Ministry of Health (Ghana Health Service)

The Ministry of Health plays a key role in ensuring adequate public health education. The study findings indicate that disposal of healthcare waste is not restricted to hospital settings. To promote awareness about SMW, the content of public messages should emphasize among others: (i) unwanted medicines, sharps and potentially infectious items confer some hazardous properties on household waste, (ii) the continuous deposit of untreated healthcare waste in landfills poses a hazard to our ecosystem; (iii) eliminating hazardous waste helps to achieve a cleaner waste stream, from which resource recovery is desirable and (iv) waste segregation and diversion can minimize potential hazards.

To avert harm from inappropriately discarded sharps waste, provision of free or highly subsidized mini-safety boxes for household use at chemical shops and pharmacies and supplied when sharps are purchased is essential. Supervised public collection systems should exist where stored sharps can be deposited for appropriate disposal. Other receptacles can be improvised such as rigid containers with tight fitting lids such as empty disinfectant containers and metal cans. Where possible, door to door pick up can be arranged at district level using local transport options such as an enclosed ‘Borla taxi’. This is a motorized cart which has the advantage of access to routes that cannot be plied by the larger refuse trucks. However, when used for this purpose, it should not be used for other purposes and it should meet standard safety recommendations. Collection from the source should be the preferred option as it is more convenient for households. Transporting safety boxes can be cumbersome for households, may encourage non-compliance and result in disposal at unauthorized sites.

Collection points can be established with trained chemical shop vendors (retail drug stores) for storage of unwanted medicines and mini-safety boxes returned by the public. A single multi-compartment vehicle can transfer them from the drug vendors to appropriate treatment and disposal facilities. Pick up from collection points to final disposal should be regular and monitored to avoid diversion for illegitimate purposes. Joint monitoring teams representing the Environmental Protection Agency, Ministry of Health, Ghana Health Service, Pharmacy Council, Ghana Police Force and respective MMDAs should conduct periodic checks on collection points and final disposal sites. Capacity building of vendors should include appropriate containerization and safety measures. This should be led by the Pharmacy Council in collaboration with Licensed Chemical Seller Association and Environmental Protection Agency.

To improve injury surveillance and primary care, hotlines can be created to facilitate communication between infection control experts at regional and tertiary hospitals and frontline providers in sub-districts. Public health nurses and community health workers which are closer to the community should be trained to conduct preliminary assessments. Public health nurses can initiate prophylaxis and make appropriate referrals where required.

### Recommendations to other stakeholders in management of solid medical waste

**Fig. 1:**
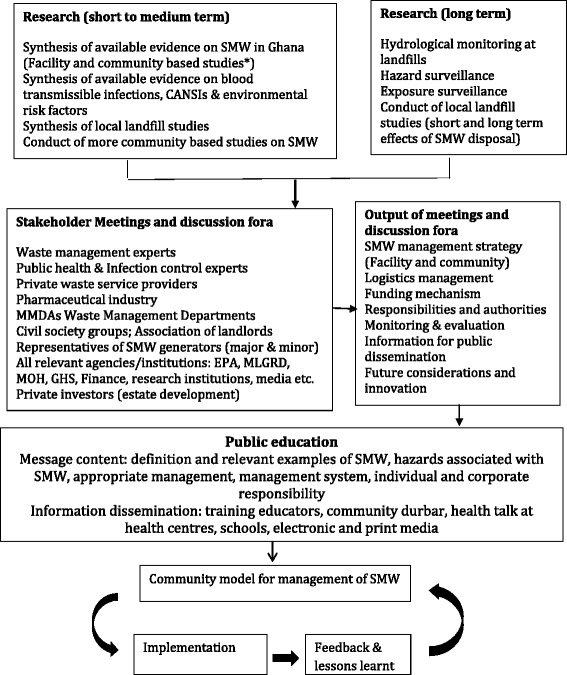
Steps in development of a community model for management of solid medical waste in households

**Fig. 2:**
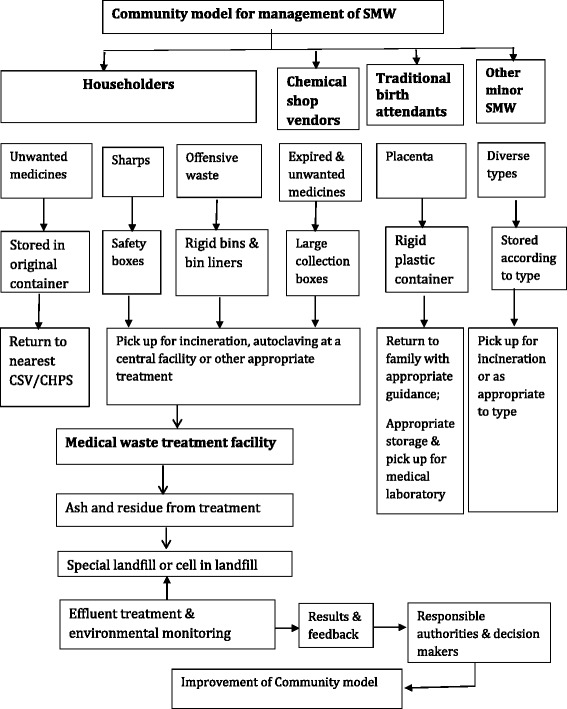
A hypothetical model for community management of solid medical waste in households

Further research is required to identify the best options for management of household medical waste. For instance, the social acceptability of diverting placentae for the production of useful derivatives for therapeutic purposes needs to be explored. If found acceptable, ethical requirements of informed consent should be integrated. The role of the media and civil society groups is essential in public education and conveying public response regarding implementation. Legislation which makes it unlawful to discard waste from healthcare activities in the household and community in an unsafe manner is required. The various recommendations are summarized in the schematic diagrams below (Figs. 1 & 2).

Limitations in the survey include recall bias although the question about harm from SMW discarded at home was restricted to one month. One month was chosen to allow time for the possibility of such an occurrence as such events were not expected to be a common occurrence. However, the possibility exists that respondents may not recall events especially if they do not perceive any danger, limitation of function or activity from such an event and if it did not translate to medical expenses. The predominance of females was due to the fact that most interviews were conducted in the evenings in order to ensure family members were present at home and females were often those available as the male members returned much later in the day. The classification of SMW was adapted to suit respondents’ understanding of relevant waste categories rather than a strict dependence on known categories of SMW in hospital settings. The study design precludes assumptions of causality but offers the opportunity for further research. The results are valid for Ga South Municipal Assembly and cannot be generalized to other districts except where contextual similarities exist.

## Conclusion

Disposal practices indicate that the majority of unwanted medicines and sharps are discarded in household bins as found in previous studies. This has potentially hazardous implications for the environment and human health since the waste is untreated and may end up in open dumps or landfills. The backyard burning of blood soaked items (especially sanitary items) is a source of air pollution in households where this is practiced. A waste stream analysis conducted on household waste in the study area would validate the disposal practices reported here and this is the subject of a forthcoming paper. In spite of the low rate of harm reported in households and the community, elimination of preventable harm due to this potentially hazardous waste is the goal and should be promoted. Presently, minimum public guidance to divert waste medicines and harmful sharps from the environment can be developed using the recommendations discussed in this paper. Future direction will evolve from additional research.

## Abbreviations

95% CI: 95% confidence intervals
SMW: Solid medical waste

## References

[1] Congress, U. S. Issues in Medical Waste Management - Background Paper. (OTA-BP-O-49). Washington, DC.: U.S. Government Printing Office; 1988.

[2] Coker, A., Sangodoyin, A., Sridhar, M., Booth, C., Olomolaiye, P., & Hammond, F. Medical waste management in Ibadan, Nigeria: obstacles and prospects. Waste Management (New York, N.Y.). 2009;29(2): 804–11. 10.1016/j.wasman.2008.06.040 18835151

[3] Hossain MS, Santhanam A, Norulaini NN, Omar AM (2011). Clinical solid waste management practices and its impact on human health and environment–a review. Waste Manag.

[4] Subratty AH, Hassed Nathire ME (2005). (2005). A survey of home generated medical waste in Mauritius. Int J Environ Health Res.

[5] Rutala WA, Mayhall CG (1992). Medical waste. Infection Control and Hospital Epidemiology : The Official Journal of the Society of Hospital Epidemiologists of America.

[6] Gold K (2011). Analysis: the impact of needle, syringe, and lancet disposal on the community. Journal of Diabetes Science and Technology.

[7] Chinbuah MA, Kager PA, Abbey M, Gyapong M, Awini E, Nonvignon J (2012). Impact of community management of fever (using antimalarials with or without antibiotics) on childhood mortality: a cluster-randomized controlled trial in Ghana. AmJTrop Med Hyg.

[8] Majumdar A, Sahoo J, Roy G, Kamalanathan S (2015). Improper sharp disposal practices among diabetes patients in home care settings: need for concern?. Indian journal of endocrinology and metabolism.

[9] Oyewole A, Sapp J, Wilson B, Oyewole O (2014). Potential environmental risks from home healthcare-generated municipal solid waste in Texas. International Journal of Business, Humanities and Technology.

[10] Miyazaki M, Imatoh T, Une H (2007). The treatment of infectious waste arising from home health and medical care services: present situation in Japan. Waste Manag.

[11] Glassmeyer ST, Hinchey EK, Boehme SE, Daughton CG, Ruhoy IS, Conerly O (2009). Disposal practices for unwanted residential medications in the United States. Environ Int.

[12] Koshy S (2013). Disposal of unwanted medications: throw, bury, burn or just ignore?. Int J Pharm Pract.

[13] Sasu S, Kümmerer K, Kranert M (2012). Assessment of pharmaceutical waste management at selected hospitals and homes in Ghana. Waste Management & Research : The Journal of the International Solid Wastes and Public Cleansing Association, ISWA.

[14] Kang’ethe SM (2008). Clinical waste management in the context of Kanye community home-based care programme. Botswana African Journal of AIDS Research.

[15] Osowiki J, Curtis N (2014). Question 2: a pointed question: is a child at risk following a community acquired needlestick injury?. Arch Dis Child.

[16] Garcia-Agar O, Vall O (1997). Hepatitis B virus infection from a needlestick. Pediatr Infect Dis J.

[17] Butsashvili M, Kamkamidze G, Kajaia M, Kandelaki G, Zhorzholadze N (2011). Circumstances surrounding the community needle-stick injuries in Georgia. J Community Health.

[18] Res S, Bowden FJ (2011). Acute hepatitis B infection following a community-acquired needlestick injury. J Inf Secur.

[19] Alvim Ferraz MCM, Afonso SAV (2003). Dioxin emission factors for the incineration of different medical waste types. Arch Environ Contam Toxicol.

[20] Ephraim PI, Ita A, Eusebius IO (2013). Investigation of soils affected by burnt hospital wastes in Nigeria using PIXE. Spring.

[21] Abor PA, Bouwer A (2008). Medical waste management practices in a southern African hospital. International Journal of Health Care Quality Assurance.

[22] Mesdaghinia A, Naddafi K, Mahvi AH, Saeedi R (2009). (2009). Waste management in primary healthcare centres of Iran. Waste Management & Research : The Journal of the International Solid Wastes and Public Cleansing Association, ISWA..

[23] GSS. Census 2010 Summary report of final results. Accra, Ghana, 2012.

[24] Watson, J. How to Determine Sample Size: Tipsheet #60. 2001. 14.

[25] Dohoo I, Martin W, Stryhn H (2012). Sampling. In: methods in epidemiologic research.

[26] Haiduven D, Ferrol S (2004). Sharps injuries in the home health care setting: risks for home health care workers. AAOHN J.

[27] Olowokure B, Duggal H, Armitage L (2003). The disposal of used sharps by diabetic patients living at home. Int J Environ Health Res.

[28] Longe E, Williams A (2006). A preliminary study of medical waste management in Lagos metropolis, Nigeria. Iranian Journal of Environmental Health Science & Engineering.

[29] WHO. Management of Solid Health-Care Waste at Primary Health-Care Centres: A Decision-Making Guide. Geneva, Switzerland: WHO Press, World Health Organization, 2005.

[30] Tong AYC, Peake BM, Braund R (2011). Disposal practices for unused medications around the world. Environ Int.

[31] Slack R, Gronow J, Voulvoulis N (2004). Hazardous components of household waste. Crit Rev Environ Sci Technol.

[32] Auta A, Omale S, Shalkur D, Abiodun AH (2011). Unused medicines in Nigerian households: types and disposal practices. J Pharmacol Pharmacother.

[33] Chen G, den Braver MW, van Gestel CA, van Straalen NM, Roelofs D (2015). Ecotoxicogenomic assessment of diclofenac toxicity in soil. Environ Pollut.

[34] Kimani D, Kamau R, Ssempijja V, Robinson K, Oluoch T, Njeru M (2014). Medical injection use among adults and adolescents aged 15 to 64 years in Kenya: results from a national survey. J Acquir Immune Defic Syndr.

[35] Jason J (2013). Community-acquired, non-occupational needlestick injuries treated in US emergency departments. Journal of Public Health.

[36] Chalupka SM, Markkanen P, Galligan C, Quinn M (2008). Sharps injuries and Bloodborne pathogen exposures in home health care. AAOHN J.

[37] Rogowska-Szadkowska D, Chlabicz S (2010). Transmission of HIV through needlestick injuries in the community setting. HIV & AIDS Review.

[38] Valavanidis A, Iliopoulos N, Gotsis G, Fiotakis K (2008). (2008). Persistent free radicals, heavy metals and PAHs generated in particulate soot emissions and residue ash from controlled combustion of common types of plastic. J Hazard Mater.

